# The Role of Potassium and KUP/KT/HAK Transporters in Regulating Strawberry (*Fragaria* × *ananassa* Duch.) Fruit Development

**DOI:** 10.3390/plants14142241

**Published:** 2025-07-20

**Authors:** José A. Mercado-Hornos, Claudia Rodríguez-Hiraldo, Consuelo Guerrero, Sara Posé, Antonio J. Matas, Lourdes Rubio, José A. Mercado

**Affiliations:** 1 Instituto de Hortofruticultura Subtropical y Mediterránea “La Mayora” (IHSM-UMA-CSIC), Universidad de Málaga, 29071 Málaga, Spain; ja-mercado@uma.es (J.A.M.-H.); claudia.rodriguez.hi@gmail.com (C.R.-H.); cguerreror@uma.es (C.G.); sarapose@uma.es (S.P.); antoniojmatas@uma.es (A.J.M.); 2 Departamento de Botánica y Fisiología Vegetal, Universidad de Málaga, 29071 Málaga, Spain; lrubio@uma.es

**Keywords:** *Fragaria*, potassium transport, fruit firmness, fruit development, KUP/KT/HAK transporters, cytosolic potassium

## Abstract

Potassium is the most abundant macronutrient in plants, participating in essential physiological processes such as turgor maintenance. A reduction in cell turgor is a hallmark of the ripening process associated with fruit softening. The dynamic of K^+^ fluxes in fleshy fruits is largely unknown; however, the reallocation of K^+^ into the apoplast has been proposed as a contributing factor to the decrease in fruit turgor, contributing to fruit softening. High-affinity K^+^ transporters belonging to the KUP/HT/HAK transporter family have been implicated in this process in some fruits. In this study, a comprehensive genome-wide analysis of the KUP/KT/HAK family of high-affinity K^+^ transporters in strawberry (*Fragaria* × *ananassa* Duch.) was conducted, identifying 60 putative transporter genes. The chromosomal distribution of the FaKUP gene family and phylogenetic relationship and structure of predicted proteins were thoroughly examined. Transcriptomic profiling revealed the expression of 19 FaKUP genes within the fruit receptacle, with a predominant downregulation observed during ripening, particularly in *FaKUP14*, *24* and *47*. This pattern suggests their functional relevance in early fruit development and turgor maintenance. Mineral composition analyses confirmed that K^+^ is the most abundant macronutrient in strawberry fruits, exhibiting a slight decrease as ripening progressed. Membrane potential (E_m_) and diffusion potentials (E_D_) at increasing external K^+^ concentrations were measured by electrophysiology in parenchymal cells of green and white fruits. The results obtained suggest a significant diminution in cytosolic K^+^ levels in white compared to green fruits. Furthermore, the slope of change in E_D_ at increasing external K^+^ concentration indicated a lower K^+^ permeability of the plasma membrane in white fruits, aligning with transcriptomic data. This study provides critical insights into the regulatory mechanisms of K^+^ transport during strawberry ripening and identifies potential targets for genetic modifications aimed at enhancing fruit firmness and shelf life.

## 1. Introduction

Ripening of fleshy fruits is a complex process involving extensive genetic and biochemical changes that make the fruits more attractive to animals for seed dispersal [1]. These changes involve, among others, the production of pigments, accumulation of soluble sugars, release of volatile compounds, and flesh softening. The postharvest shelf life of fleshy fruits is mainly determined by the rate of fruit softening, which affects storage duration, shipping viability, susceptibility to bruising and pathogens, spoilage throughout the supply chain, and consumer acceptance [2]. Over-softening represents a significant source of economic losses, particularly for rapidly softening fruits such as strawberries.

Fruit softening is primarily attributed to modifications of the primary cell wall structure and middle lamellae, reducing intercellular adhesion and cell wall strength. This process is driven by the activation of genes encoding cell wall remodeling enzymes, such as polygalacturonase, pectate lyase, and endoglucanases [3,4]. Gene editing of some of these genes significantly improved postharvest shelf life in fruits like tomato (*Solanum lycopersicum* L.) and strawberry (*Fragaria* × *ananassa* Duch.) [5,6]. Besides cell wall remodeling, loss of cell turgor due to transpirational water loss and/or solute accumulation in the apoplast also contributes to firmness reduction, particularly in berry fruits [7,8]. In grape (*Vitis vinifera* L.), turgor loss is an early ripening event that precedes the activation of key genes encoding cell wall-modifying enzymes [9]. The genetic basis of this reduction in turgor is largely unknown, but solute membrane transporters are likely involved.

Potassium is the most abundant cation in plant tissues, including fruits, and plays a crucial role in maintaining cell turgor [10]. Five major multi-gene families encode K^+^ transport systems: shaker-like K^+^ channels, tandem-pore K^+^ (TPK) channels, KUP/KT/HAK transporters, HKT transporters, and cation–proton antiporters [11]. In strawberry, turgor changes during fruit ripening have received limited attention. However, the significant role of K^+^ channels in fruit ripening has recently been demonstrated. Thus, an ABA-induced potassium channel gene (*FaKAT1*), which shows increased expression in ripe fruit, was identified [12]. Transient suppression of this gene delayed strawberry ripening, reduced transcript levels of some ripening-related genes, and decreased ABA accumulation. Similarly, transient silencing of the ubiquitous two-pore K^+^ channel gene *FaTPK1* delayed strawberry fruit ripening [13]. TPK channels, which are involved in vacuolar K^+^ homeostasis, may promote sucrose accumulation, potentially acting as a ripening signal [14].

In addition to K^+^ channels, high-affinity H^+^:K^+^ symport transporters (HAK) belonging to the KUP/KT/HAK transporter family are also involved in K^+^ fluxes. These genes are predominantly expressed in roots but also found in shoots under K^+^ starvation [15]. In grape, two KUP/KT/HAK-type genes are highly expressed in the skin during early fruit development, with declining expression after the onset of ripening [16]. These transporters may contribute to K^+^ uptake during early berry growth when sugars have not accumulated in the vacuole and potassium serves as the primary osmoticum [16]. In peach (*Prunus persica*) fruit, 16 putative KUP/KT/HAK transporters were identified and the expression of some of them was related to fruit growth and firmness [17,18]. The KUP/KT/HAK gene *AcKUP2* in kiwifruit (*Actinidia chinensis* Planch.) is ethylene-regulated during ripening [19]. In *Fragaria*, the K^+^ transporter gene family was studied in the diploid strawberry *Fragaria vesca* [20]. The expression of the KUP/KT/HAK genes identified was analyzed in shoots and roots under K^+^ deficiency but not in fruits. The silencing of polygalacturonase genes in transgenic strawberry reduced fruit softening, and, interestingly, a putative potassium transporter like-5 gene, *FvH4_6g30550.1*, was upregulated in transgenic fruits [21]. It was suggested that the induction of the potassium transporter could contribute to maintaining the solute potential and the turgor pressure of fruit cells, contributing to the increased firmness observed in transgenic fruits alongside less cell wall disassembly.

This research aimed to investigate the role of K^+^ fluxes and the KUP/KT/HAK K^+^ transporters in strawberry fruit ripening. A genome-wide analysis of the KUP/KT/HAK family members was performed in the *F.* × *ananassa* genome, and their expression during fruit development was evaluated. Additionally, electrophysiological experiments were conducted to measure plasma membrane potential to estimate K^+^ permeability and cytosolic K^+^ levels in fruits at different developmental stages.

## 2. Results

### 2.1. Identification of KUP/KT/HAK in Strawberry

A BLAST search against the *F*. × *ananassa* genome v1.0.a2 (https://www.rosaceae.org/; accessed on 3 October 2023) using 13 Arabidopsis KUP/KT/HAK protein sequences as queries identified 94 mRNA sequences. Pfam domain searches using the HMMER web service (https://www.ebi.ac.uk/Tools/hmmer/; accessed on 17 October 2023) revealed 60 proteins containing the PF02705 domain typical of K^+^ transporters and exceeding 500 amino acids in length. These were designated FaKUP1 to FaKUP60 (Supplementary Table S1). Protein lengths ranged from 566 to 980 amino acids, with molecular weights ranging from 63.9 to 109.2 kDa and isoelectric points varying from 5.2 to 9.5. A common feature of the tridimensional models of different KUP/KT/HAK proteins is the presence of a hydrophobic core containing a variable number of transmembrane (TM) domains, 8 to 14 depending on the predicted service [22]. In strawberry, structural predictions indicated that all putative K^+^ transporters had transmembrane helices; 56 contained 10–14 TM domains, 3 had 8–9 TM domains, and the largest protein had 18 TM domains.

**Figure 1:**
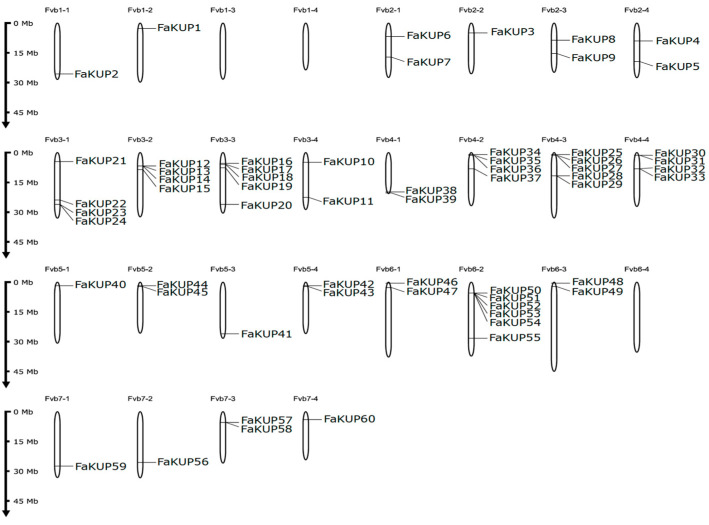
Chromosomal localization of KUP/KT/HAK genes in the *F*. × *ananassa* genome.

The chromosomal distribution of the FaKUP genes in the *F*. × *ananassa* genome is shown in Figure 1. Chromosomes 3-1, 3-2, 3-3, 4-2, 4-3, 4-4, and 6-2 harbored most of the transporter genes, while chromosomes 1-3, 1-4, and 6-4 lacked any KUP/KT/HAK family member. In chromosomes with a higher density of K^+^ transporters, such as chromosome 6-2, genes were often clustered.

### 2.2. Phylogenetic and Motif Analysis of Predicted KUP/KT/HAK Proteins

**Figure 2:**
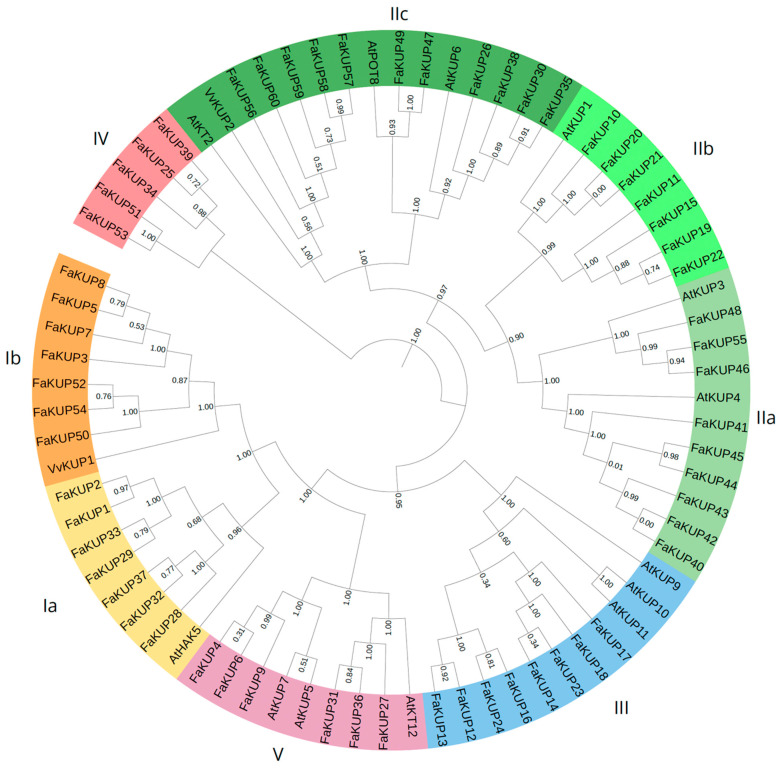
Phylogenetic tree of the KUP/KT/HAK proteins identified in *F.* × *ananassa* genome. A total of 13 *Arabidopsis* proteins and 2 *Vitis* proteins were also included (all belonging to the KUP/KT/HAK transporter family). The maximum likelihood method was used to infer the relationship between sequences. Statistical support of each branch is provided. Genes used in the analysis are listed in Supplementary Table S2.

Phylogenetic analysis of KUP/KT/HAK proteins from strawberry and Arabidopsis (*Arabidopsis thaliana*) was conducted. Two grape proteins putatively involved with berry development (VvKUP1 and VvKUP2 [16]) were also included in the analysis. The resulting tree identified five major clades (I to V) as described by [23] (Figure 2). Clades I and II were subdivided into two and three subgroups, respectively. As previously described, Arabidopsis KUP/KT/HAK proteins were distributed across clades I, II, III, and V [23]. VvKUP1 belonged to clade Ib while VvKUP2 was part of clade IIc. Most strawberry FaKUP proteins clustered in clades I (14 proteins; 23.3% of total *Fragaria* sequences) and II (27 proteins, 45%). Clade IV contained only *Fragaria* proteins.

Motif analysis identified seven conserved sequences, ranging from 29 amino acids (motifs 5 and 7) to 50 amino acids (motif 4) (Table 1). Motifs were located near the N-terminus (motifs 1 and 7), the C-terminus (motifs 2 and 3), or within the central region (motifs 3, 4, and 5) (Supplementary Figure S1). Two strawberry sequences, FaKUP2 and FaKUP3, lacked N-terminal motifs, while the largest protein, FaKUP13, contained motifs near the C-terminus. The sequence GVVYGDLGTSPLY, which is highly conserved among different KUP/KT/HAK proteins [24], was found in motif 7.

### 2.3. Expression of KUP/KT/HAK in Strawberry Fruits

**Figure 3:**
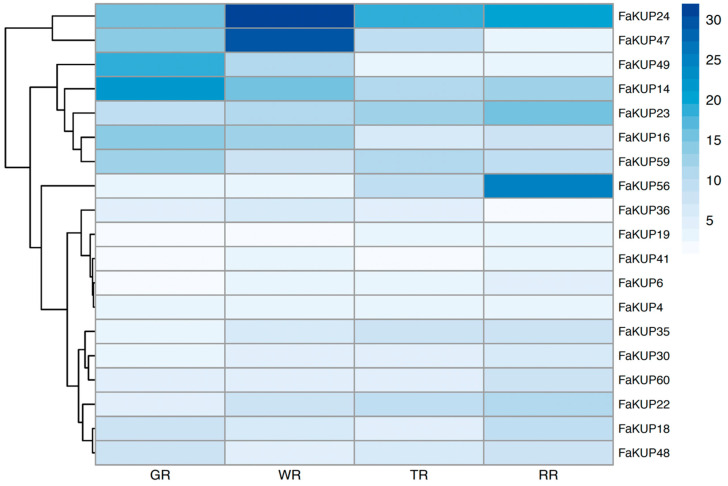
Expression profile of FaKUP genes during strawberry fruit development, cv. ‘Camarosa’. The expression levels (transcript per million, TPM) were obtained from [25]. GR: green receptacle; WR: white receptacle; TR: turning receptacle; RR: red receptacle.

The expression of KUP/KT/HAK genes in strawberry fruit receptacles at different developmental stages was obtained from the RNAseq study performed in the genotype ‘Camarosa’ [25]. Only 19 out of the 60 genes analyzed were expressed in this tissue (Figure 3), with the highest expression corresponding to *FaKUP24* and *FaKUP47*. Both genes reached their highest expression level at the white stage and later showed decreased expression during ripening. Four additional genes (*FaKUP49*, *FaKUP14*, *FaKUP16* and *FaKUP59*) were downregulated from green to ripe stages. Only *FaKUP56* and *FaKUP23* were upregulated during ripening. The remaining KUP/KT/HAK genes showed lower expression levels.

**Figure 4:**
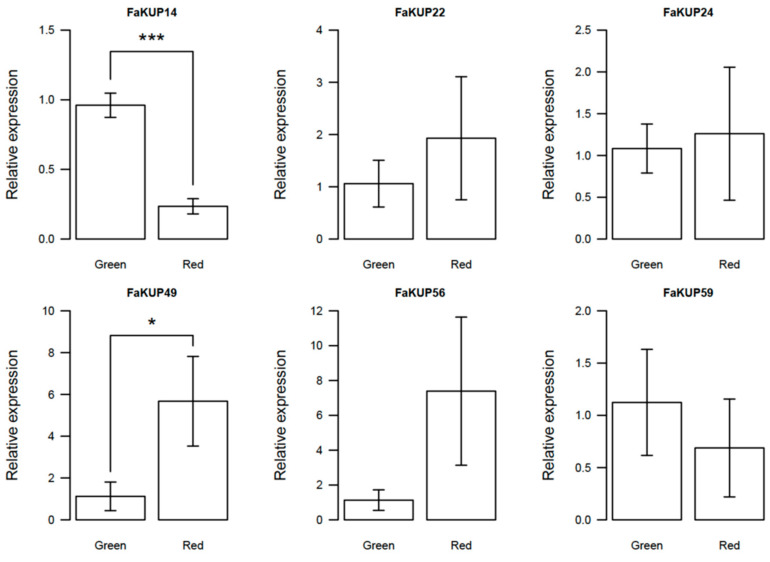
Expression of some selected FaKUP genes measured by quantitative RT-PCR in strawberry fruits, cv. ‘Chandler’, at two developmental stages, green unripe and red ripe. Mean separation by Student’s *t*-test. Differences statistically significant at * *p* = 0.05 or *** *p* = 0.001.

The values of expression of FaKUP genes were also searched in a RNAseq of ripe fruits cv. ‘Chandler’ [26]. Both sets of expression data were significantly correlated (Pearson coefficient of 0.81, significant at *p* = 0.01; Supplementary Figure S2 and Table S3). Some FaKUP genes with high expression values in both RNAseq studies were further analyzed by qRT-PCR in green unripe and red ripe fruits of the ‘Chandler’ cultivar (Figure 4). *FaKUP14* was significantly downregulated in red fruit. *FaKUP22*, *FaKUP24*, and *FaKUP59* maintained similar expression levels in green unripe and ripe fruit. *FaKUP49* and *FaKUP56* were upregulated in red fruits (Figure 4).

The promoter regions of KUP/KT/HAK genes expressed in fruits were also analyzed. A total of four motifs were found to be enriched in the promoter region of putative KUP/KT/HAK genes that were downregulated during ripening (*FaKUP14*, *FaKUP16*, *FaKUP24*, *FaKUP47*, and *FaKUP59*) (Supplementary Table S4). These motifs were associated with the binding site of transcription factors belonging to MYB and ERF/DREB families. By contrast, no enriched motifs were found in the promoter regions of putative KUP/KT/HAK genes that were upregulated during ripening (*FaKUP23*, *FaKUP49*, and *FaKUP56*). Finally, a motif associated with the MYB transcription factor family was found to be enriched in the promoter region of the rest of the putative KUP/KT/HAK genes that were expressed in fruits.

### 2.4. Mineral Composition of Strawberry Fruits

The mineral composition of the strawberry receptacle at the green unripe and red ripe stages was analyzed by ICP (Table 2). Potassium was the most abundant element on a dry weight basis. The content of all macroelements decreased during ripening, although the decline in K^+^ was not statistically significant. Sodium content was the highest among the micronutrients. The content of most microelements decreased during ripening, but only the reduction in iron content was statistically significant. Total nitrogen decreased significantly from 15.1 ± 0.3 g·kg^−1^ in unripe fruit to 8.9 ± 1.3 g·kg^−1^ in ripe fruit (*p* = 0.05), while carbon content remained unchanged. The C:N:H ratios were 45.9:1.5:5.1 in unripe fruit and 45.3:0.9:5.4 in ripe fruit.

### 2.5. Cytosolic K^+^ Estimation by Electrophysiological Thecnique.

**Figure 5:**
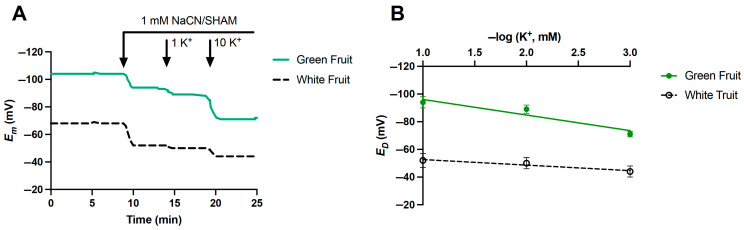
Membrane potential (E_m_, mV) of parenchymal cells from green and white fruits. (**A**) Traces are representative time course recordings of single parenchymal cells for each fruit type (n =10). Downward arrows indicate the onset of 1 mM NaCN and 1 mM SHAM followed by the external K^+^ concentration increments. (**B**) Curve fit of diffusion potential (E_D_) of parenchymal cells from green and white fruits at different external KCl concentrations. Values are mean ± SD of at least 3 independent fruits per developmental stage.

Electrophysiological measurements in strawberry fruits were conducted to estimate cytosolic K^+^. Green unripe fruits, white fruits at the onset of ripening, and fully red fruits were used. Due to the soft texture of fully ripe fruits, reliable measurements could not be obtained as cells broke down upon microelectrode insertion. Plasma membrane potential (E_m_) was significantly lower in green than in white fruits (−104.7 ± 14.9 mV vs. −68.3 ± 5.7 mV, respectively; *p* = 0.05). Apparent diffusion potential values (E_D_) were obtained after the addition of 1 mM NaCN and 1 mM SHAM to the assay media, containing 0.1 mM KCl, to inhibit ATP synthesis and H^+^ pumping (Figure 5). Under these conditions, cytosolic K^+^ serves as a charge balance that counteracts membrane depolarization [27]. As expected, E_D_ values became less negative as the external K^+^ concentration increased, especially in white fruit (Figure 5), suggesting that cytosolic K^+^ should be lower in white than in green fruit. In addition, the slope of E_D_ changes in response to external K^+^ was higher in green fruit than in white fruit (11 mV/pK vs. 4 mV/pK, respectively), indicating lower plasma membrane permeability to K^+^ in white fruits. Assuming equilibrium, cytosolic K^+^ concentrations were estimated using the Nernst equation at 10 mM KCl, resulting in 161.9 ± 16.5 mM in green fruit and 60.5 ± 23.0 mM in white fruit (*p* = 0.01).

## 3. Discussion

Potassium is an important macronutrient in plants, participating in essential functions both at the cell and plant levels, including growth processes, turgor maintenance, and phloem transport [28]. Along this line, enrichment of nutrient solutions with K^+^ has been shown to improve overall strawberry plant growth and fruit quality, although the response is cultivar-specific [29]. In fruits, K^+^ serves as an osmoticum during initial cell growth and expansion, and later during ripening, it could play a role in reducing cell turgor, leading to fruit softening [28]. This study provides insights into the role of high-affinity K^+^ transporters from the KUP/KT/HAK family and K^+^ dynamics in strawberry fruit development.

### 3.1. KUP/KT/HAK Transporters Form a Large Family in Cultivated Strawberry

KUP/KT/HAK transporters constitute one of the five major K^+^ transport systems in plants [11]. Genes encoding KT/HAK/KUP proteins have been implicated in K^+^ accumulation and fruit development in grape and peach [16,17,18]. In the diploid species *F. vesca*, one of the progenitors of *F*. × *ananassa*, 15 KUP/KT/HAK genes were identified [20], which fall within the typical range of 14–20 members for most angiosperm genomes [22]. The identification of 60 KUP/KT/HAK genes in cultivated strawberry underscores the complexity of its octoploid genome. Phylogenetic analysis revealed the distribution of these proteins into five clades, as previously described [23]. Most *Fragaria* proteins belonged to clades II and I. Although functional studies of KUP/KT/HAK transporters are scarce, it has been suggested that clade II members are associated with developmental processes, especially those related to turgor-driven cell expansion [23], while transporters involved in root high-affinity K^+^ uptake from dicot and monocot species belong to clade Ia.

Previous studies found that the sequence GVVYGDLGTSPLY is highly conserved among different KUP/KT/HAK proteins, including KUP of *Escherichia coli* [24]. This motif is located in the N-terminus at the first transmembrane segment of the protein and contains some residues essential for transporter function [30]. In our study, this sequence was found inside motif 7, and some amino acids of the GVVYGDLGTSPLY sequence were conserved in all FaKUP genes analyzed. Motif 1, also located in the N-terminus, contained additional conserved residues, highlighting its potential importance for transporter function.

### 3.2. Expression of KUP/KT/HAK Genes Declined During Strawberry Ripening

A transcriptomic study performed in ‘Camarosa’ fruits showed that only 19 out of the 60 FaKUP strawberry genes were expressed during fruit receptacle development. In general, the expression of these genes was low and, with the exception of a small number of FaKUP members, most of them were downregulated as the fruit ripened. The functional significance of FaKUP gene expression patterns was supported by qRT-PCR analysis in ‘Chandler’ fruits, which validated RNA-seq findings for most genes. *FaKUP49* was the only studied gene that showed a clearly contradictory expression pattern during ripening when comparing published RNA-seq data and RT-qPCR data. This could indicate differential expression of *FaKUP49* between cultivars or could be due to technical issues in RNA-seq. Differences in FaKUP expression patterns may indicate distinct roles for these transporters at different developmental stages. During early fruit growth, FaKUP genes may facilitate K^+^ incorporation to maintain turgor-driven cell expansion. In contrast, FaKUP genes activated during ripening may contribute to apoplastic K^+^ accumulation, leading to reduced turgor and fruit softening. Most of the FaKUP genes expressed in fruit fall in clades IIc and III. The *Vitis* KUP/KT/HAK gene *VvKUP2* associated with potassium fluxes in grape also belongs to clade IIc. Interestingly, Arabidopsis genes in clade IIc negatively regulate turgor-dependent growth of roots by mediating K^+^ efflux [31]. The two genes upregulated in ‘Chandler’ red fruits, *FaKUP49* and *FaKUP56*, also belong to clade IIc. On the other hand, a member of clade III from cotton (*Gossypium hirsutum*), *GhKT1*, has been involved in elongation, inducing the accumulation of K^+^, which allowed fiber growth driven by the increase in cell turgor [32]. *FaKUP14*, which is downregulated during ripening, is also a member of this clade.

### 3.3. K^+^ Content and Cytosol K^+^ Diminished During Fruit Ripening

Potassium dynamics during fruit development have been extensively studied in grape. In this fruit, K^+^ accumulation declined at veraison, the onset of grape ripening characterized by color, flavor, and texture changes [28]. The apoplastic solute potential drops sharply during this phase, decreasing from −0.2 MPa early in development to −1.0 MPa at veraison and −4 MPa at late ripening stages, due to the accumulation of K^+^ and other solutes [33]. In strawberry, K^+^ content was only slightly lower in ripe fruit than in green fruit on a dry weight basis. On a fresh weight basis, this difference was more pronounced due to increased water content during fruit development [34]. However, the drop in K^+^ content was less significant compared to other macronutrients, particularly calcium. It has been found that xylem functionality declines during strawberry development, and, in ripe fruits, it represents around 36% of total water inflow vs. 64% of phloem flow [35]. As K^+^ is predominantly transported by the phloem and to a lesser extent via the xylem [36], the reduced xylem contribution may partially explain the observed decrease in K^+^ content. On the other hand, the osmotic potential decreased during strawberry ripening, reaching values lower than −1 MPa at maturity [35]. Unfortunately, the contribution of the apoplastic solutes and, in particular, K^+^ to this decrease in the water potential is unknown.

Electrophysiology is the most convenient approach to study membrane transport in plants. This approach is difficult to implement in fruits mainly due to their soft texture, particularly at ripening. To our knowledge, electrophysiological studies in fruits are limited to a seminal paper reporting the membrane potential of kiwi vesicles [37]. In this research, we established the procedure for performing electrophysiological measurements in strawberry fruits. When compared with ionic diffusion equilibria, the E_m_ could be a good indicator of H^+^-ATPase activity; i.e., a negative membrane voltage largely in excess of the equilibrium voltage indicates active energy-dependent ion pumping [38]. The values of E_m_ obtained in green unripe fruits were in the range of those typically reported for plant cells; however, the membrane was significantly depolarized in white fruits. This result suggests that metabolite transport through plasmodesmata is favored over apoplastic active transport as the fruits ripen. Supporting this hypothesis, the density of plasmodesmata in strawberry fruit parenchymal cells increased in white vs. green fruits [39]. On the other hand, the results obtained indicate that membrane permeability to K^+^ as well as cytosolic K^+^ decreased during ripening, consistent with transcriptomic data showing downregulation of KUP/KT/HAK genes. Membrane depolarization and lower activity of other potassium-permeable transport systems could also contribute to the decline in cytosolic K^+^ concentration as the fruit ripens.

## 4. Materials and Methods

### 4.1. Plant Material

Strawberry plants (*Fragaria* × *ananassa* Duch.) cultivar ‘Chandler’ were obtained from runner propagation of mother plants maintained in the greenhouse at IFAPA Centro de Churriana (Málaga, Spain). Fifteen plants were grown in 22 cm pots containing a substrate mix of peat moss, sand, and perlite (volume ratio of 6:3:1). The plants were cultivated in a greenhouse under natural temperature and light conditions. Average monthly temperatures ranged from 15 °C in January to 24 °C in June. The irrigation regime was 3 L.m^−2^.day^−1^ and plants were fertilized weekly with Nitrofoska^®^ solub 12-5-30 (EuroChem Agro Iberia S.L., Barcelona, Spain). ‘Chandler’ fruits were harvested at three developmental stages: unripe (green receptacle with green achenes), white, and fully ripe (entirely red receptacle). Samples were used for electrophysiological measurements or immediately frozen in liquid nitrogen and stored at −80 °C until molecular analysis.

### 4.2. Identification of KUP/KT/HAK

The full length of the 13 KUP/KT/HAK protein sequences described in Arabidopsis were used as query sequences for a BLAST search against the *F.* × *ananassa* genome (Camarosa Genome Assembly v1.0.a2; [25]). The BLASTp tool from the Genome Database for Rosaceae (https://www.rosaceae.org/) was employed. Theoretical molecular weights and isoelectric points of the identified proteins were calculated using the R package Seqinr v4.2-8. Conserved domains and transmembrane segments were predicted using InterProScan v5.64-96.0. Proteins predicted to have a complete K^+^ transporter domain (PF02705) were retained for further analysis. In addition, as KUP/KT/HAK transporters identified in other species such as peach, diploid strawberry, and apple (*Malus domestica*) had more than 500 amino acids [23], this length criterion was also applied.

The physical location of each putative KUP/KT/HAK gene in the *F.* × *ananassa* Camarosa Genome v1.0.a2 was retrieved from the Genome Database for Rosaceae. Then, a genomic map including the location of each putative KUP/KT/HAK gene was drawn using MG2C v2.1.

### 4.3. Phylogenetic and Motif Analysis

For multiple sequence alignment, the iterative method G-INS-i of MAFFT v7 software was used with default parameters. A total of 75 protein sequences were aligned, including the 60 putative members of the KUP/KT/HAK family in *F.* × *ananassa*, 13 from *A. thaliana*, and 2 from *V. vinifera* (VvKUP1 and VvKUP2). Phylogenetic tree construction was conducted using the maximum likelihood method in PhyML via SeaView v5.0.4 with default parameters. Tree visualization and annotation were performed using iTOL v6. Conserved motifs were identified using MEME v5.5.4, with the maximum number of motifs set to seven.

### 4.4. Promoter Region Analysis

Putative high-affinity K^+^ transporters that were expressed in fruits were selected for analysis of the promoter region. The promoter region of each gene was defined as the 1000 bp upstream of the transcription start site. The program Analysis of Motif Enrichment (AME) v.5.5.8 [40] was used to identify known motifs that were enriched in these promoters with respect to control sequences (randomly created by the program by combining nucleotide fragments from the input sequences themselves). The database of known motifs used was ‘JASPAR CORE Plants and Arabidopsis 2024’. A motif was considered enriched if the adjusted *p*-value was lower than 0.05.

### 4.5. Gene Expression Analysis

Previously published transcriptomic data from the fruit receptacle of ‘Camarosa’ at various developmental stages [25] and ‘Chandler’ at the fully ripe stage [26] were analyzed to evaluate KUP/KT/HAK expression in strawberry fruit. Quantitative real-time PCR (qRT-PCR) was used to measure the expression of selected genes in ‘Chandler’ fruits at unripe and ripe stages. Total RNA was extracted from de-achened fruits following the protocol by [41]. RNA concentration and purity were assessed using a Nanodrop™ spectrophotometer (Thermo Scientific, Waltham, MA, USA) and 1% agarose gel electrophoresis. Reverse transcription was performed from 1 μg of total RNA using the QuantiTectTM Re-verse Transcription Kit (QIAGEN, Germany), according to the manufacturer’s instructions. Prior to qRT-PCR experiments, primer efficiency was evaluated to choose the best primer pairs. qRT-PCR was performed in a final reaction volume of 10 μL containing SsoAdvanced Universal SYBRTM Green Supermix 1× (BIO-RAD, Hercules, CA, USA), 0.5 μM of each specific primer, and 1 μL of diluted cDNA (1:20). Amplification reactions were carried out in a CFX Opus 384 Touch Real-Time PCR Detection System (BIO-RAD) using the following program: 95 °C for 3 min; 40 cycles at 95 °C for 10 s and 57 °C for 30 s; and a melting curve from 65 °C to 95 °C with 0.5 °C increments at 5 s intervals. The actin reference gene was used for normalization [42] and relative expression levels were calculated using the 2^−ΔΔCt^ method [43]. Primer sequences are listed in Supplementary Table S5. Three independent RNA extractions were performed per ripening stage and used as biological replicates. Three technical replicates were carried out per biological sample.

### 4.6. Mineral Composition of Strawberry Fruit

Fruits of cv. ‘Chandler’ at the unripe and ripe stages were de-achened, oven-dried at 80 °C for 72 h, and then ground into a fine powder. Mineral composition was determined by inductively coupled plasma (ICP) spectrometry. Each sample, approximately 100 mg, was digested using 3 mL of 65% HNO_3_ (Suprapure, Supelco, Switzerland) in a Milestone Ultrawave pressurized microwave digestion system at 240 °C and 40 Bar pressure. After digestion, the aliquots were diluted to a final volume of 25 mL with deionized water. Subsequently, ICP analysis was performed at the Atomic Spectrometry Unit of the Central Research Support Services (SCAI) of the University of Málaga. Quantitative analysis of Ca, Mg, and K was performed using an Optima 7300DV inductively coupled plasma–optical emission spectrometer (ICP-OES) from Perkin Elmer (Waltham, MA, USA). Quantitative analysis of P, B, Na, Mn, Cu, Fe, and Zn was performed using a Nexion 300D inductively coupled plasma–mass spectrometer (ICP-MS) from Perkin Elmer, equipped with a single quadrupole analyzer and a collision cell using helium gas. Analyses were conducted in triplicate, with each replicate comprising a pool of three fruits.

### 4.7. Electrophysiological Experiments

‘Chandler’ fruits at different developmental stages were collected and immediately prepared for electrophysiological measurements. Thin longitudinal sections of inner fruit tissue were fixed with paraffin wax on a Plexiglass perfusion chamber and covered with a coverslip. The inner space of the chamber (1.1 mL volume) was continuously perfused with basal solution (CaCl_2_ 2 mM, KCl 0.1 mM, MES 2 g/L, and Bis-Tris 0.35 g/L, pH 5.7) at a flux of approximately 10 mL min^−1^. Membrane potentials (E_m_) were measured using standard glass microelectrode techniques [44]. For measurements, single-barrel microelectrodes, connected to a high-impedance differential amplifier (FD223; World Precision Instruments, Sarasota, FL, USA), were inserted into parenchymal cells, with continuous perfusion of the assay medium. Diffusion potentials (E_D_) were recorded following the addition of 1 mM NaCN and 1 mM salicylhydroxamic acid (SHAM) to inhibit ATP synthesis and H^+^ pumping [43]. Cytosolic K^+^ was estimated by monitoring E_D_ at increasing external K^+^ concentrations (0.1, 1, and 10 mM KCl) and using the Nernst equation with E_D_ values obtained at 10 mM KCl [44,45,46,47]. Data represent average values from 3 to 6 fruits per developmental stage.

### 4.8. Statistical Analysis

Statistical analyses were performed using R software, v. 4.4.2. Mean comparisons were made using Student’s *t*-test.

## 5. Conclusions

This study provides comprehensive insights into the role of KUP/KT/HAK potassium transporters in strawberry fruit development. A total of 60 KUP/KT/HAK genes were identified in the *F.* × *ananassa* genome, revealing their structural diversity and phylogenetic relationships. The expression profiling of these genes highlighted their differential regulation during fruit ripening, with several genes downregulated as the fruit transitioned from green to ripe stages. These expression patterns suggest a possible role for KUP/KT/HAK transporters in regulating potassium fluxes that influence fruit firmness and softening. The electrophysiological experiments further demonstrated a decline in cytosolic K^+^ concentrations during ripening, consistent with the decreased expression of KUP/KT/HAK genes. The low membrane permeability to K^+^ in ripening fruit cells indicates potential changes in ion transport dynamics that contribute to cell turgor loss and fruit softening. Overall, the findings of this research underscore the importance of potassium transporters in fruit physiology and development. Future studies involving functional characterization of key KUP/KT/HAK genes through gene editing or overexpression strategies could provide deeper insights into their specific roles in fruit ripening and quality enhancement. This knowledge could inform breeding programs aimed at developing strawberry varieties with improved postharvest characteristics and shelf life.

## Figures and Tables

**Table 1:** Sequences of the motifs found in KUP/KT/HAK proteins. Amino acids in red are conserved in all sequences. The shaded amino acids correspond to the conserved sequence described by [24].

Motif Name	Sequence
Motif 1	SLIFWTLTLIPLLKYVFIVLRABDNGEGGTFALYSLJCRHA
Motif 2	CIKSVPVPKVPPEERFLVGRVGPKEYRMFRCIARYGYKD
Motif 3	IVASQAIISATFSIIKQSLALGCFPRVKVV
Motif 4	WISLGGIVLCITGTEAMFADLGHFSVRSIQIAFTCVVYPCLVLAYMGQAA
Motif 5	YIPEINWILMILCLAVTIGFRDTKQIGNA
Motif 6	LGIVRVPGIGLVYTELVSGIPAIFSHFVTNLPAFHSVVVFV
Motif 7	LAYQSLGVVYGDLGTSPLYVYKSTFSGGI

**Table 2:** Mineral composition of strawberry fruit at unripe and ripe stages. Means with different letters indicate significant differences by Student’s *t*-test at *p* = 0.05.

		Unripe	Ripe
Macroelements (g·kg^−1^ dw)	Potassium	19.9 ± 0.6 a	16.3 ± 3.1 a
	Calcium	4.4 ± 1.7 a	1.3 ± 0.2 b
	Magnesium	3.1 ± 0.4 a	1.5 ± 0.2 b
	Phosphorus	3.3 ± 0.1 a	2.5 ± 0.3 b
Microelements (mg·kg^−1^ dw)	Sodium	281.3 ± 73.9 a	175.2 ± 3.2 a
	Iron	55.5 ± 3.7 a	42.8 ± 4.8 b
	Manganese	18.7 ± 6.5 a	10.7 ± 1.4 a
	Boron	18.4 ± 4.3 a	12.2 ± 2.2 a
	Zinc	13.7 ± 4.5 a	7.1 ± 1.1 a
	Copper	1.5 ± 0.09 a	1.6 ± 0.3 a

## Data Availability

All data generated or analyzed during this study are included in this published article [and its Supplementary Information files].

## Supplementary Materials

The following supporting information can be downloaded at https://www.mdpi.com/article/10.3390/plants14142241/s1, Figure S1: Motif distribution in KUP/KT/HAK family proteins; Figure S2: Correlation between FaKUP gene expression levels in ‘Camarosa’ and ‘Chandler’ red receptacle; Table S1: List of KUP/KT/HAK genes identified in *Fragaria* × *ananassa;* Table S2: List of genes used in the phylogenetic analysis; Table S3: Values of FaKUP gene expression in fruit red receptacle from RNA-seq studies performed in cultivars ‘Camarosa’ and ‘Chandler’; Table S4: Motifs found enriched in promoter region of KUP/KT/HAK expressed in fruit; Table S5: Primer sequences used in this research.
