# Supplementary material for: The Role of Potassium and KUP/KT/HAK Transporters in Regulating Strawberry (Fragaria × ananassa Duch.) Fruit Development
Source: Plants (Basel). 2025 Jul 20;14(14):2241. doi: 10.3390/plants14142241 (PMC12300644; doi:10.3390/plants14142241)
Supplement: Supplementary file 1 [file plants-14-02241-s001.zip › SFigure.pdf]

The Role of Potassium and KUP/KT/HAK Transporters in Regulating Strawberry (Fragaria × ananassa Duch.) Fruit Development

José A. Mercado-Hornos[1], Claudia Rodríguez-Hiraldo[1], Consuelo Guerrero[1], Sara Posé[1], Antonio J. Matas[1], Lourdes Rubio[2], and José A. Mercado[1*]

[1]Instituto de Hortofruticultura Subtropical y Mediterránea "La Mayora" (IHSM-UMA-CSIC), Departamento de Botánica y Fisiología Vegetal, Universidad de Málaga, 29071 Málaga, Spain

[2]Departamento de Botánica y Fisiología Vegetal, Universidad de Málaga, 29071 Málaga, Spain

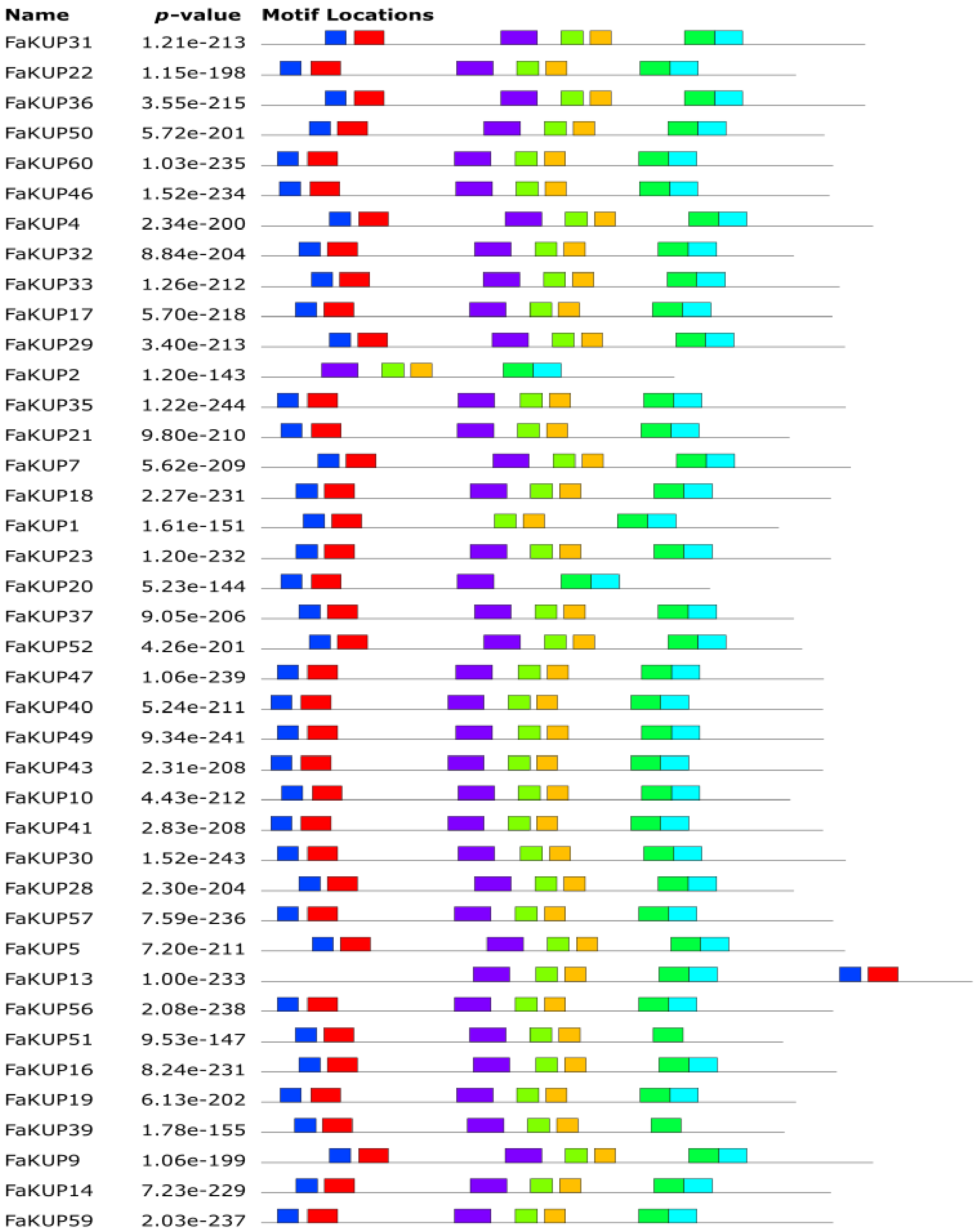
Name
p-value
Motif Locations
FaKUP31 1.21e-213
FaKUP22 1.15e-198
FaKUP36 3.55e-215
FaKUP50 5.72e-201
FaKUP60 1.03e-235
FaKUP46 1.52e-234
FaKUP4 2.34e-200
FaKUP32 8.84e-204
FaKUP33 1.26e-212
FaKUP17 5.70e-218
FaKUP29 3.40e-213
FaKUP2 1.20e-143
FaKUP35 1.22e-244
FaKUP21 9.80e-210
FaKUP7 5.62e-209
FaKUP18 2.27e-231
FaKUP1 1.61e-151
FaKUP23 1.20e-232
FaKUP20 5.23e-144
FaKUP37 9.05e-206
FaKUP52 4.26e-201
FaKUP47 1.06e-239
FaKUP40 5.24e-211
FaKUP49 9.34e-241
FaKUP43 2.31e-208
FaKUP10 4.43e-212
FaKUP41 2.83e-208
FaKUP30 1.52e-243
FaKUP28 2.30e-204
FaKUP57 7.59e-236
FaKUP5 7.20e-211
FaKUP13 1.00e-233
FaKUP56 2.08e-238
FaKUP51 9.53e-147
FaKUP16 8.24e-231
FaKUP19 6.13e-202
FaKUP39 1.78e-155
FaKUP9 1.06e-199
FaKUP14 7.23e-229
FaKUP59 2.03e-237

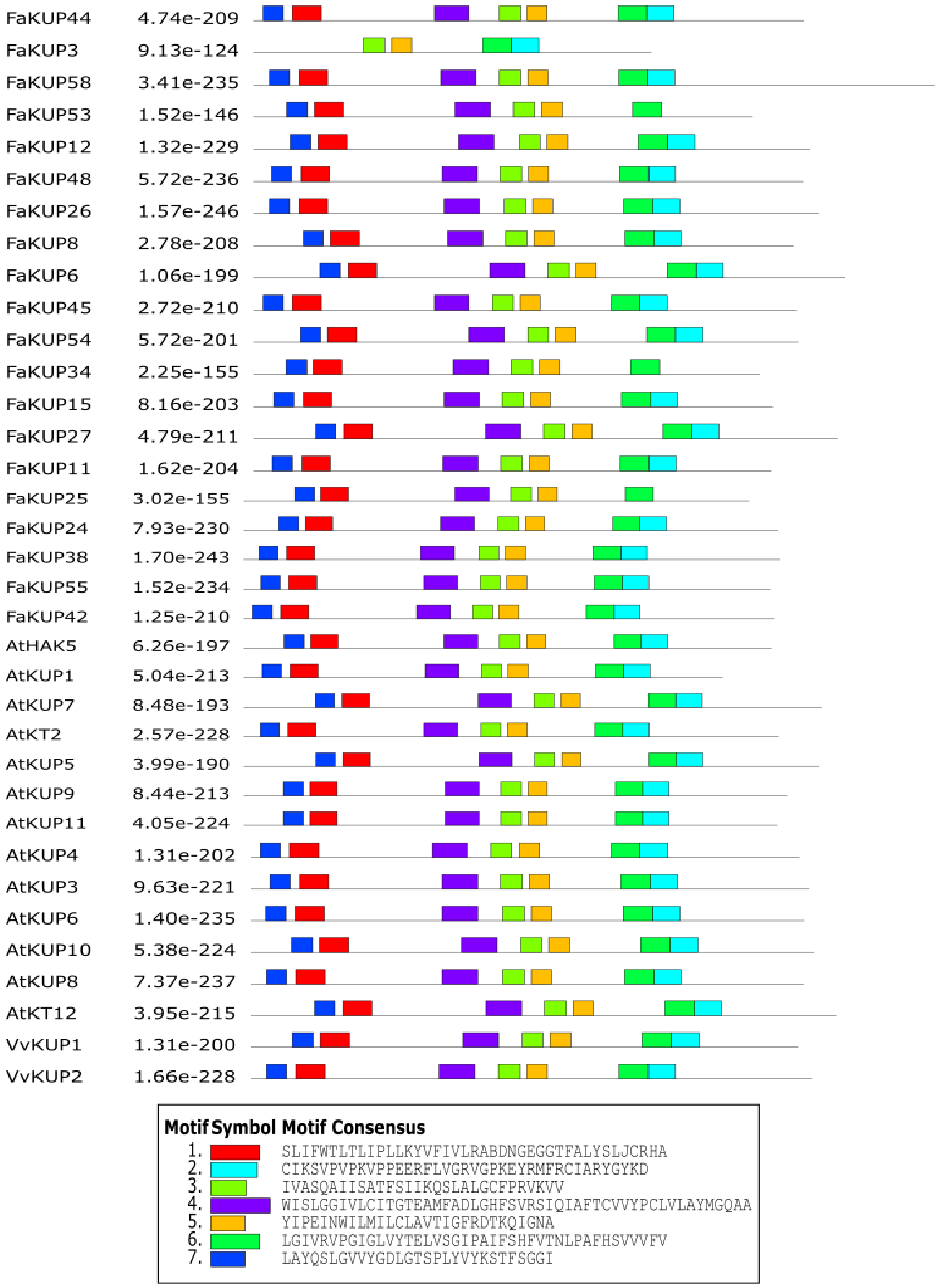

Supplementary Figure S1. Motif distribution in KUP/KT/HAK family proteins

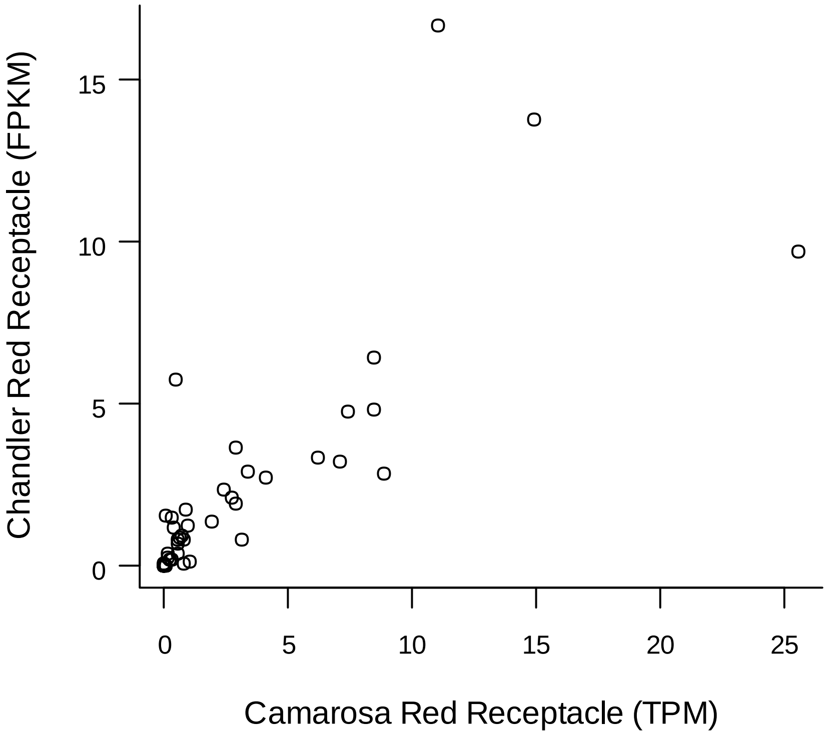

Supplementary Figure S2. Correlation between gene expression levels of FaKUP genes in RNAseq studies of 'Camarosa' (Liu et al., 2021) and 'Chandler' (Ric-Varas et al., 2024) red receptacle. TPM: transcript per million; FPKM: fragments per kilobase of transcript sequence per million base pairs sequenced.
